# IL-23 induces human osteoclastogenesis via IL-17 *in vitro*, and anti-IL-23 antibody attenuates collagen-induced arthritis in rats

**DOI:** 10.1186/ar2297

**Published:** 2007-09-23

**Authors:** Toru Yago, Yuki Nanke, Manabu Kawamoto, Takefumi Furuya, Tsuyoshi Kobashigawa, Naoyuki Kamatani, Shigeru Kotake

**Affiliations:** 1 Institute of Rheumatology, Tokyo Women's Medical University, 10-22 Kawada-cho, Shinjuku-ku, Tokyo 162-0054, Japan

## Abstract

This study demonstrates that IL-23 stimulates the differentiation of human osteoclasts from peripheral blood mononuclear cells (PBMC). Furthermore, *in vivo *blockade of endogenous IL-23 activity by treatment with anti-IL-23 antibody attenuates collagen-induced arthritis in rats by preventing both inflammation and bone destruction. IL-23 induced human osteoclastogenesis in cultures of PBMC in the absence of osteoblasts or exogenous soluble-receptor activator of NF-kappaB ligand (RANKL). This IL-23-induced osteoclastogenesis was inhibited by osteoprotegerin, anti-IL-17 antibody, and etanercept, suggesting that RANKL, IL-17, and TNF-alpha are involved. In addition, we found the ratio of production levels of IL-17 to those of IFN-gamma from activated human T cells was elevated at 1 to 10 ng/ml IL-23. The inductive effect of IL-17 and the inhibitory effect of IFN-gamma on osteoclastogenesis indicate that the balance of these two cytokines is particularly important. We also demonstrated that IL-23 administered at a later stage significantly reduced paw volume in rats with collagen-induced arthritis, in a dose-dependent manner. Furthermore, anti-IL-23 antibody reduced synovial tissue inflammation and bone destruction in these rats. These findings suggest that IL-23 is important in human osteoclastogenesis and that neutralizing IL-23 after onset of collagen-induced arthritis has therapeutic potential. Thus, controlling IL-23 production and function could be a strategy for preventing inflammation and bone destruction in patients with rheumatoid arthritis.

## Introduction

Rheumatoid arthritis is a chronic inflammatory disease characterized by the destruction of articular cartilage and bone [1]. Our group and another have detected osteoclasts in synovial tissues [2] and eroded bone surfaces [3], suggesting that osteoclastic bone resorption is involved in the pathogenesis of rheumatoid arthritis (RA).

Furthermore, levels of inflammatory cytokines such as TNF-α, IL-6, and IL-1 are elevated in synovial fluids of patients with RA [4,5], and the cytokines promote bone resorption by inducing the differentiation or activation of osteoclasts [2,6,7]. It is well known that attenuating the activity of inflammatory cytokines in patients with RA inhibits bone resorption and destruction.

IL-23, which was recently identified as a heterodimeric, proinflammatory cytokine and new member of the IL-12 family [8], is secreted by antigen-presenting cells. IL-23 is composed of p19 and p40 subunits and shares a common p40 subunit with IL-12 [8]. IL-23 signals through the IL-23 receptor complex, which is composed of the IL-12 receptor β chain and the IL-23 receptor [9]. IL-23 was initially described as a cytokine able to induce the expression of IFN-γ in human CD45RO-positive (memory) T cells and to activate memory T cells to secrete inflammatory cytokines including IFN-γ and IL-17 [8,10]. Furthermore, it is reported that recombinant human (rh)IL-23 upregulates the production of IFN-γ, IL-17, and IL-10 in activated human naïve T cells [11]. In models of T helper type 1 (Th1) differentiation of human T cells, it was initially proposed that IL-23 acts later than IL-12 and maintains Th1 commitment by its preferential action on memory T cells [12-14].

In animal studies, it is reported that IL-23-deficient (IL-23 p19^-/-^) mice are resistant to experimental autoimmune encephalomyelitis (EAE), whereas IL-12 (p35)-deficient mice are still susceptible to inflammation [15]. Murphy and colleagues reported that mice with collagen-induced arthritis (CIA) and IL-23 deficiency (IL-23 p19^-/-^) are completely resistant to the development of joint and bone pathology and that IL-23 is required for the induction of joint inflammatory mediators including IL-17 and TNF-α [16]. Furthermore, transgenic mice constitutively overexpressing IL-23 p19 develop spontaneous severe multi-organ inflammation with elevated levels of TNF-α [17]. These findings suggest that IL-23 has a pivotal role in the establishment and maintenance of inflammatory autoimmune diseases. In addition, some reports have established the idea of a critical function for the IL-23–IL-17 pathway in some autoimmune diseases and emphasize the importance of understanding the origins of development of IL-17 effector cells [10,18].

IL-17 is a proinflammatory cytokine secreted by activated T cells [19] or neutrophils [20]. We have reported that IL-17 levels in synovial fluids are significantly higher in patients with RA than in patients with osteoarthritis and that IL-17 stimulates osteoclast differentiation by inducing the expression of receptor activator of NF-κB ligand (RANKL) via a mechanism involving the synthesis of prostaglandin E_2 _in osteoblasts *in vitro *[21]. In addition, we reported that IL-17 directly stimulates human osteoclastogenesis from human monocytes alone, via the TNF-α or RANK–RANKL pathway [22]. Recently, some groups have reported that IL-17 is also important in joint destruction in animal models and in patients with RA [23-25]. It is therefore indicated that IL-23 is involved in osteoclastic bone resorption, at least in part via the IL-17 pathway, and that IL-23 is important in the progression of arthritis. However, the direct effect of IL-23 on human osteoclastogenesis from peripheral blood mononuclear cells (PBMC) and the role of anti-IL-23 antibody in CIA in rats remain unclear.

In the present study we examined the direct role of IL-23 in osteoclastogenesis by using cultures of human PBMC. Furthermore, to clarify the role of IL-23 antibody in the later stage of CIA, rats with CIA were treated with anti-IL-23 antibody at a later stage after the onset of clinical arthritis.

## Materials and methods

### Reagents

rhIL-23 and anti-IL-17 antibody were purchased from R&D Systems Inc. (Minneapolis, MN, USA). Goat polyclonal anti-IL-23 antibody was purchased from Santa Cruz Biotechnology (Santa Cruz, CA, USA). Recombinant human macrophage-colony stimulating factor (M-CSF; Leukoprol) was obtained from Yoshitomi Pharmaceutical (Osaka, Japan). Recombinant human soluble RANKL (sRANKL) was obtained from PeproTech (London, UK). Microbeads for immunopurification were obtained from Miltenyi Biotec (Auburn, CA, USA). Anti-human CD51/61 mAb was purchased from BD Bioscience Pharmingen (San Diego, CA, USA). Osteoprotegerin (OPG) was a gift from Sankyo Pharmaceutical (Tokyo, Japan), and etanercept was purchased from Takeda Pharmaceutical (Tokyo, Japan).

### Culture system for osteoclastogenesis in the absence of osteoblasts

Human peripheral blood was obtained from the buffy coat fraction from healthy volunteers (Japanese Red Cross Society, Tokyo, Japan) after this study had been approved by the Institutional Review Board. PBMC were isolated by centrifugation over Histopaque 1077 (Sigma, St Louis, MO, USA) density gradients, washed, and resuspended at 1.3 × 10^6 ^cells/ml in α-MEM (Gibco BRL, Gaithersburg, MD, USA) supplemented with 10% fetal bovine serum (JRH Biosciences, Lenexa, KS, USA). PBMC were cultured for 3 days in 48-well plates (5 × 10^5 ^cells/0.3 ml per well; Corning, NY, USA) in the presence of M-CSF (100 ng/ml) and various concentrations of rhIL-23 (R&D Systems Inc., Minneapolis, MN, USA). In some experiments, we simultaneously added OPG (250 ng/ml), etanercept (0.01 μg/ml), or anti-IL-17 antibody (5 μg/ml). Adherent PBMC were used as monocytes in the culture system. After non-adherent cells had been removed, adherent PBMC, as described above, were cultured in the presence of M-CSF for 7 days. Culture medium was replaced every 3 days with fresh medium supplemented with the agents described above. Osteoclast formation was evaluated by immunohistochemical staining for vitronectin receptors after 7-day culture. As a negative control, human PBMC were cultured for the first 3 days in the presence of M-CSF and then adherent cells were further cultured with M-CSF alone for 7 days. As a positive control, human PBMC were cultured for the first 3 days in the presence of M-CSF and then adherent cells were further cultured with M-CSF and sRANKL (100 ng/ml) for 7 days.

### Determination of osteoclast characteristics

Adherent cells were fixed and stained for vitronectin receptor [26]. For immunohistochemical staining, adherent cells cultured for 7 days were fixed with cold methanol/acetone (50:50, v/v) for 10 minutes. Samples were then incubated with monoclonal antibodies against vitronectin receptor αvβ3 (CD51/61). Bound antibodies were revealed with biotinylated secondary antibodies, avidin–biotin-conjugated peroxidase, and a diaminobenzidine substrate kit (Histofine; Nichirei Co., Tokyo, Japan). Tartrate-resistant acid phosphatase (TRAP) activity was detected as described previously [21]. Pit formation assay was performed with Osteologic^® ^(BD Biosciences, San Jose, CA, USA). Phagocytosing activity was detected by phagocytes of fluoresbrite YG microspheres^® ^(Poly Sciences, Inc., Warrington, PA, USA).

### IL-17 and IFN-γ measurement from human T cells stimulated by IL-23

Human CD3-positive T cells were separated from PBMC obtained from the same volunteers by magnetic cell sorting (Miltenyi Biotec, Sunnyvale, CA, USA) and cultured in 96-well plates (10^5 ^cells/0.2 ml per well; Iwaki, Tokyo, Japan) containing α-MEM supplemented with 10% fetal bovine serum. We verified that CD3-positive T cells were purified to 98% after magnetic cell sorting. Plates were coated overnight with anti-CD3 mAb (0.1 μg/ml; Beckman Coulter, Fullerton, CA, USA) and then washed; anti-CD28 mAb (2 μg/ml; Beckman Coulter) was then added to each well. At the beginning of cell culture, rhIL-23 was added at 1, 3, or 10 ng/ml. After 48 hours, supernatants were collected to determine cytokine levels. Amounts of IL-17 and IFN-γ in the supernatants were measured with ELISA kits (R&D Systems Inc., Minneapolis, MN, USA) in accordance with the manufacturer's instructions.

### Animals

A total of 28 seven-week-old female DA/Slc rats (Nihon SLC, Hamamatsu, Japan) were housed in a temperature-controlled room (21 to 26°C) with a 12-hour alternating light/dark cycle. Animals were given rat chow (Oriental Kobo, Tokyo, Japan) and water *ad libitum *before and throughout the experiments. Animal studies were approved by the Institutional Review Board.

### Induction of collagen-induced arthritis

Animals were handled 2 weeks before experiments and every 2 to 3 days throughout the study. Food and fluid intake as well as body weight were monitored. Arthritis was induced by immunizing with type II collagen (CII; Anthrogen-CIA™Collagen; Chondrex, LLC, Redmond, WA, USA). Dissolved CII (0.3%, 2 ml) was emulsified with 3 ml of Freund's incomplete adjuvant and 1 ml of saline. The final concentration of CII was 1 mg/ml. Rats were immunized intradermally in the tail with 200 μl of emulsion on days 0 and 7 [27]. Onset of arthritis usually occurred during the window of days 12 to 14. Treatment with anti-IL-23 antibody was initiated on day 14, 10 days after the first clear onset of clinical signs of arthritis as demonstrated by ankle joint swelling.

### Experimental protocol

Arthritis was induced in 16 female DA/Slc rats, which received 3 μg ('low') or 6 μg ('high') anti-IL-23 antibody (Santa Cruz Biotechnology) intraperitoneally on alternate days from day 14 (later stage, after CIA onset) up to day 23. Eight other rats received an equal volume of sterile phosphate-buffered saline. Arthritis was not induced in four female DA/Slc rats, acting as normal controls. A total of 28 rats were killed on day 28.

### Assessment of arthritic damage

Disease progression was monitored from the induction of arthritis (day 0) until day 28, when the rats were killed with intraperitoneal pentobarbital. Four indices of arthritis activity were used, and joint swelling in both ankles was measured every 3 to 4 days by plethysmometry (TK101CMP; Muromachi-kikai, Tokyo, Japan). Arthritis score was measured every 3 to 4 days by inflammation of paws (0, normal; 1, mild swelling and erythema of digits or ankles; 2, moderate swelling and erythema of digits or ankles; 3, marked swelling of paws including digits; 4, severe swelling and erythema with limited motion in many joints). Rats were killed on day 28, and both ankles were removed for histologic and radiographic examination to assess joint damage. Radiographic examination was performed in all rats. After radiography, paraffin sections were prepared for histological analysis.

### Statistical analysis

Data were analyzed with the Mann–Whitney *U *test, Student's *t *test, and Welch's *t *test (Stat View^®^; Abacus Concepts, Berkeley, CA, USA). *P *values less than 0.01 or 0.05 were considered significant. All values are represented as means and SD.

## Results

### IL-23 directly induces osteoclastogenesis from human PBMC

**Figure 1:**
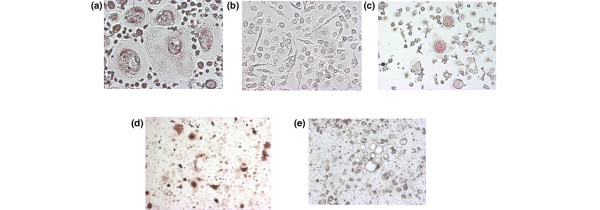
Formation of human osteoclasts from PBMC induced by rhIL-23 (1.0 ng/ml) and M-CSF. As negative and positive controls, peripheral blood mononuclear cells (PBMC) were cultured with macrophage-colony stimulating factor (M-CSF) alone during the first 3 days; as a negative control, adherent cells were then cultured with only M-CSF **(a)**, and as a positive control they were cultured with M-CSF plus soluble receptor activator of NF-κB ligand (sRANKL; 100 ng/ml) **(b) **for the last 7 days. Human osteoclasts induced from PBMC by recombinant human (rh)IL-23 (1.0 ng/ml) and M-CSF were detected by staining with tartrate-resistant acid phosphatase (TRAP) **(c) **and immunohistological staining by vitronectin receptor αvβ3 (CD51/61) **(d)**. **(e) **Osteoclasts induced from PBMC by rhIL-23 (1.0 ng/ml) were also evaluated functionally by pit formation on Osteologic^®^. Original magnification ×100.

**Figure 2:**
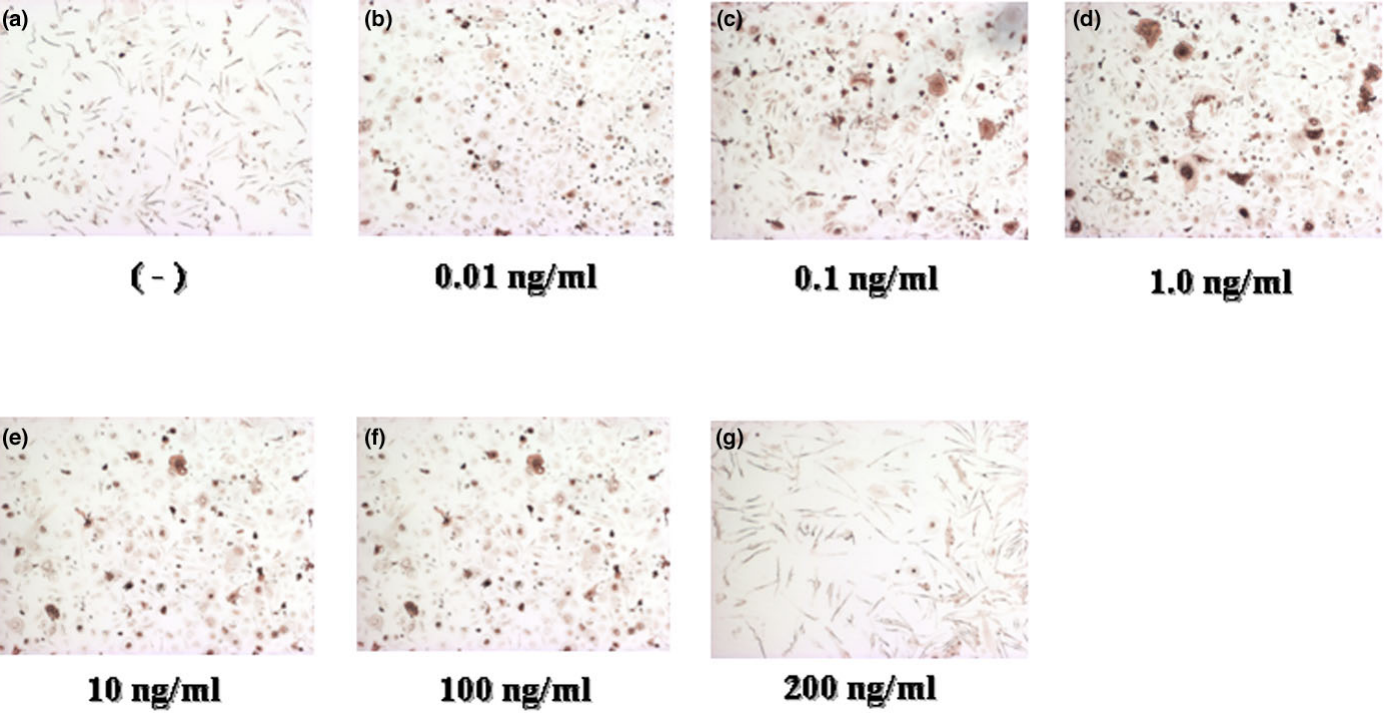
IL-23-induced formation of human osteoclasts from peripheral blood mononuclear cells (PBMC). PBMC were cultured in the presence of macrophage-colony stimulating factor (M-CSF) and recombinant human (rh)IL-23 (0.01, 0.1, 1.0, 10, 100, or 200 ng/ml) during the first 3 days. Adherent cells were then cultured with M-CSF alone during the last 7 days (days 4 to 10 **(a–g) **as indicated). Original magnification ×100.

**Figure 3:**
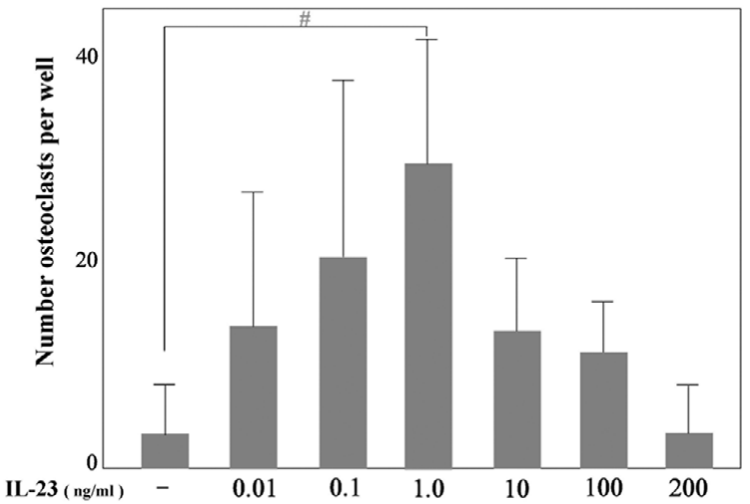
IL-23-induced osteoclastogenesis from cultured human peripheral blood mononuclear cells. A variable concentration of recombinant human (rh)IL-23 (0.01, 0.1, or 1 ng/ml) was present during the first 3 days (days 0 to 4). After 10 days, osteoclasts positive for anti-vitronectin receptor antibody were counted. Data are expressed as means and SD for triplicate cultures. Experiments were repeated three times with similar, significant results; ^#^*P *= 0.002 (Student's *t *test).

To investigate whether rhIL-23 induces osteoclastogenesis, human PBMC were cultured with M-CSF and rhIL-23 for 3 days; after non-adherent cells had been removed, adherent cells were cultured with M-CSF alone for 7 days. Compared with a negative control (Figure 1a) and a positive control (Figure 1b) stained for TRAP, multinuclear cells formed by rhIL-23 and M-CSF showed TRAP activity (Figure 1c), CD51 expression (Figure 1d), and the ability to form resorption pits on Osteologic^® ^(Figure 1e). The multinuclear cells therefore showed the functions and properties of authentic osteoclasts. Stimulation with rhIL-23 for 3 days increased the number of vitronectin receptor (CD51)-positive multinuclear cells. As shown in Figure 2, rhIL-23 increased the number of CD51-positive osteoclasts in a dose-dependent manner even in the absence of osteoblasts or exogenous sRANKL. The effect of osteoclastogenesis induced by IL-23 was maximal at 1.0 ng/ml rhIL-23 (Figure 3). In contrast, rhIL-23 did not induce osteoclastogenesis in a culture of human monocytes alone (data not shown). These findings suggested that T cells were required for IL-23-induced human osteoclastogenesis from PBMC.

### IL-23-induced osteoclastogenesis is inhibited by OPG, anti-IL-17 antibody and etanercept

**Figure 4:**
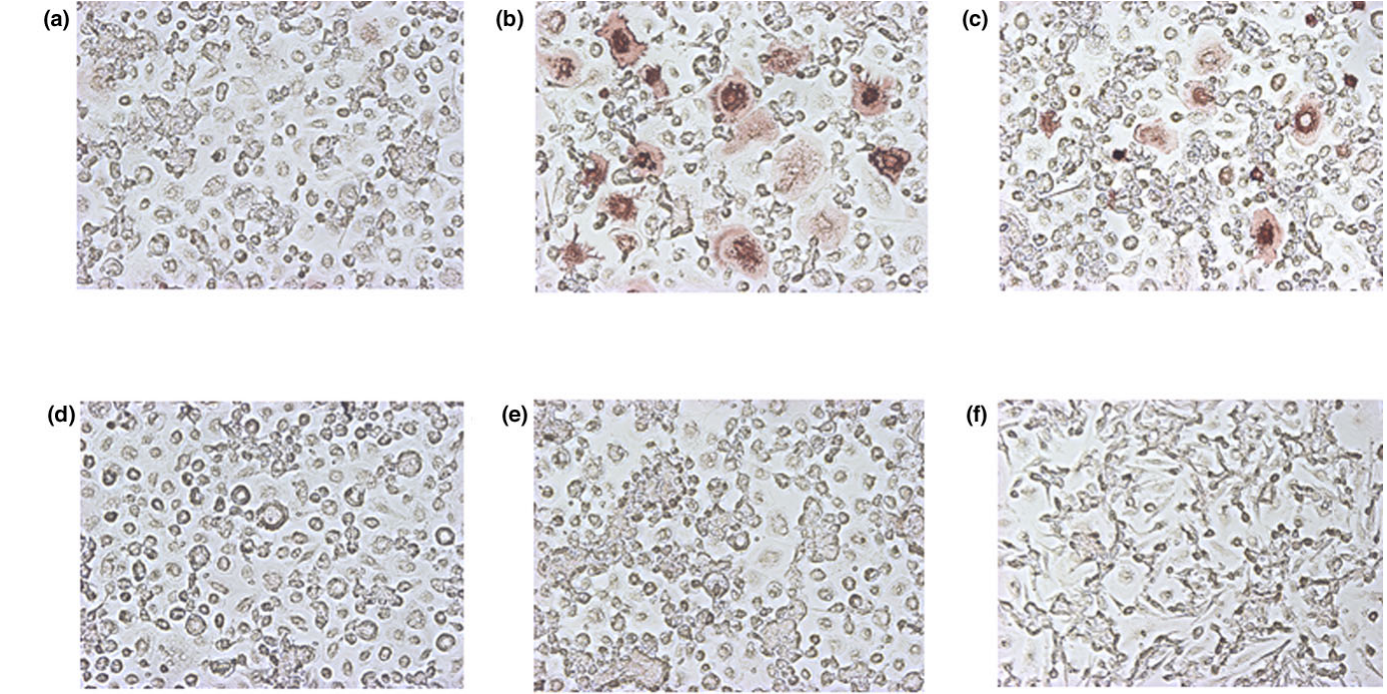
Inhibition of IL-23-induced osteoclastogenesis by osteoprotegerin, anti-IL-17 antibody, and etanercept. Peripheral blood mononuclear cells (PBMC) were cultured during the first 3 days with macrophage-colony stimulating factor (M-CSF) and recombinant human (rh)IL-23 (1.0 ng/ml) **(c)**. At the same time, osteoprotegerin (OPG, 250 ng/ml) **(d)**, anti-IL-17 antibody (5 μg/ml) **(e)**, or etanercept (0.01 μg/ml) **(f) **was added with rhIL-23 (1.0 ng/ml). Adherent cells were cultured with M-CSF alone during the last 7 days (days 4 to 10 **(c–f)**). As controls, PBMC were cultured with M-CSF alone during the first 3 days, after which adherent cells were cultured with M-CSF only **(a) **(negative control) or with soluble receptor activator of NF-κB ligand (sRANKL; 100 ng/ml) **(b) **(positive control). Osteoclasts were detected by immunohistological staining for vitronectin receptor αvβ3 (CD51/61). Original magnification ×100.

To investigate factors influencing IL-23-induced osteoclastogenesis, we used OPG, anti-IL-17 antibody, or etanercept as a TNF-α inhibitor with rhIL-23 (1.0 ng/ml) in cultured PBMC. As shown in Figure 4, IL-23-induced osteoclastogenesis was inhibited by OPG (Figure 4d), anti-IL-17 antibody (Figure 4e), and etanercept (Figure 4f). These findings suggested that IL-23 induced osteoclastogenesis via a pathway including RANK–RANKL, IL-17, and TNF-α.

### The ratio of production levels of IL-17 to those of IFN-γ from activated human T cells by IL-23 is dose-dependently elevated without significant changes in IL-17 production

**Figure 5:**
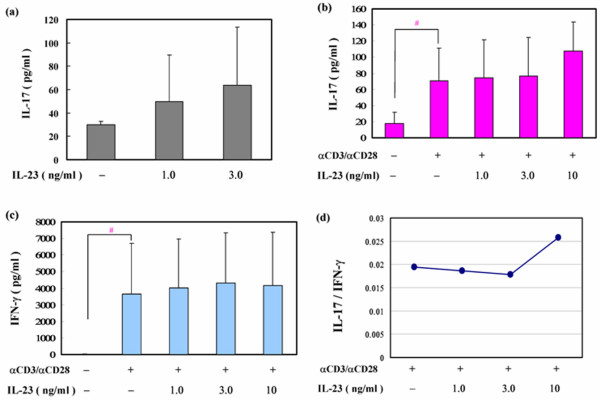
IL-23-induced IL-17 production by activated human T cells in a dose-dependent manner. **(a) **Human CD3-positive T cells were cultured in the absence or presence of recombinant human (rh)IL-23 (1.0 or 3.0 ng/ml). After 48 hours, secretion of IL-17 in the cell culture supernatant was assayed by ELISA. Results for one representative donor are presented here and are expressed as means and SD. **(b) **ELISA measurement of IL-17 production by human CD3-positive T cells treated for 48 hours with various concentrations of rhIL-23 (1.0, 3.0, or 10 ng/ml) in the presence or absence of plate-bound anti-CD3 and anti-CD28, for five representative donors. Data are expressed as means and SD. ^#^*P *= 0.009 versus non–activated T cells (Mann–Whitney *U *test). **(c) **ELISA measurement of IFN-γ production by human CD3-positive T cells treated for 48 hours with various concentrations of rhIL-23 (1.0, 3.0, or 10 ng/ml) in the presence or absence of plate-bound anti-CD3 and anti-CD28, for four representative donors. Data are expressed as means and SD. ^#^0.02 versus non–activated T cells **(**Mann–Whitney *U *test). **(d) **Ratio of the production levels of IL-17 to those of IFN-γ (IL-17/IFN-γ) calculated from levels of IL-17 or IFN-γ produced from activated CD3-positive T cells at each concentration of rhIL-23 (0, 1, 3, or 10 ng/ml).

To clarify the pathways underlying IL-23-induced osteoclastogenesis, we investigated proinflammatory cytokine production by human T cells stimulated by IL-23. As shown in Figure 5a, IL-23 induced IL-17 production in human non–activated T cells in a dose-dependent manner; however, there were no significant changes among different IL-17 levels. IL-23 also induced IL-17 production in human activated T cells, and the levels of IL-17 secreted by activated T cells were significantly higher than those secreted by non–activated T cells (Figure 5b). However, levels of IL-17 secreted by activated T cells were not significantly changed by stimulation with IL-23 (Figure 5b). As well as the production of IL-17, IL-23 also induced that of IFN-γ by activated human T cells, and the levels of IFN-γ secreted by activated T cells were significantly higher than those secreted by non–activated T cells; however, the levels of IFN-γ secreted by activated T cells were not significantly changed by stimulation with IL-23 (Figure 5c). The ratio of production levels of IL-17 to levels of IFN-γ peaked at 10 ng/ml IL-23 (Figure 5d).

### Time course of collagen-induced arthritis

**Figure 6:**
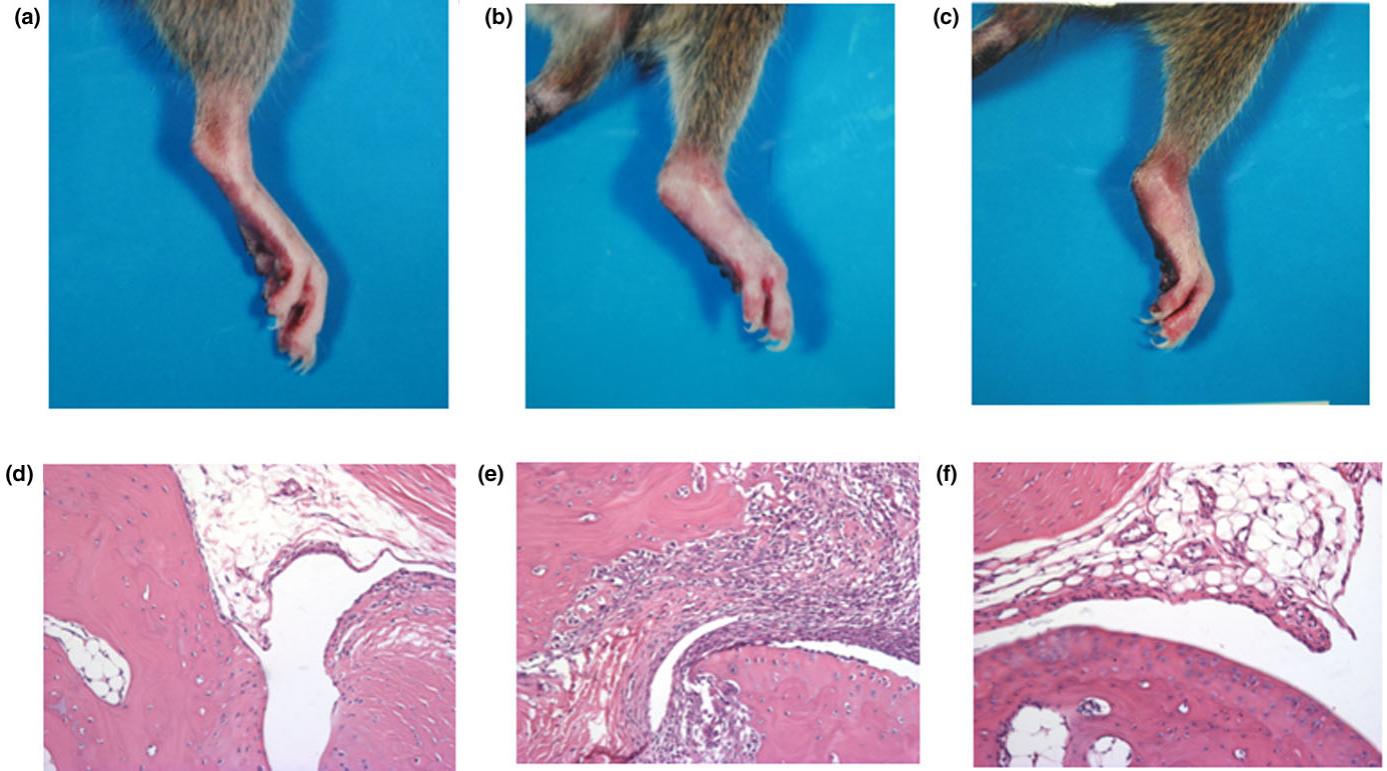
Variation in degree of inflammation of rat paws after treatment with vehicle or IL-23 blockade. **(a–c) **Photographs showing paws of rats with collagen-induced arthritis (CIA) that were treated with vehicle or anti-IL-23 antibody on day 21. **(a) **Control rats. **(b) **Rats with CIA that were treated with vehicle. **(c) **Rats with CIA that were treated with anti-IL-23 antibody (3.0 μg) from day 14. **(d–f) **Slides stained with hematoxylin and eosin representing synovial tissues obtained from right paws on day 28. **(d) **Control rats. **(e) **Rats with CIA that were treated with vehicle. **(f) **Rats with CIA that were treated with anti-IL-23 antibody (3.0 μg) from day 14. Original magnification ×100.

**Figure 7:**
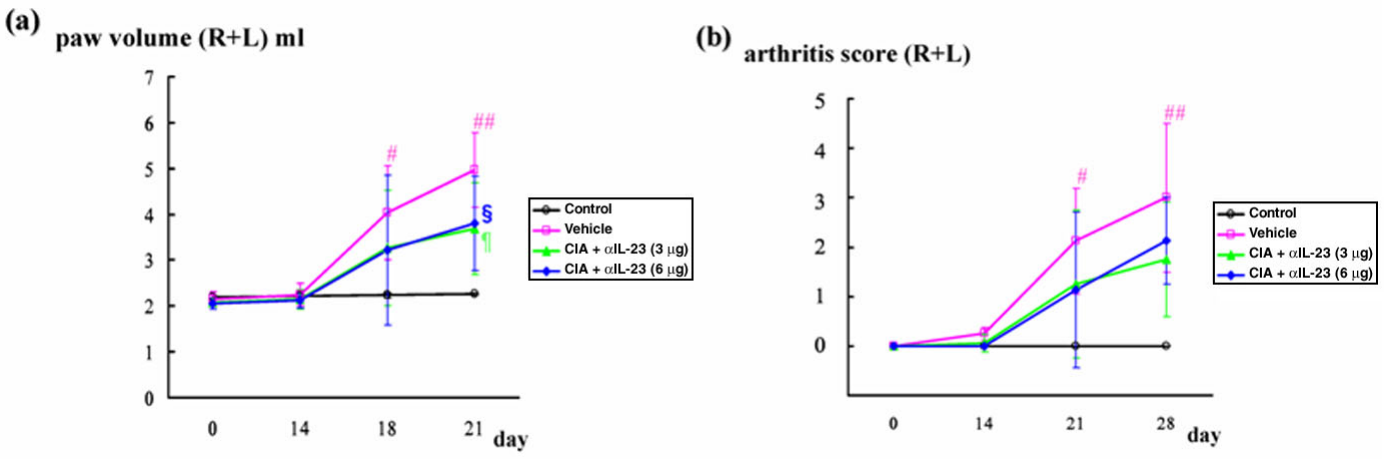
Variation in paw volume in rats with CIA treated with vehicle or IL-23 blockade. **(a) **Paw volumes of control rats, vehicle-treated rats with collagen-induced arthritis (CIA), and rats with CIA that were treated with anti-IL-23 antibody, from day 0 to day 21. Open squares, rats with CIA treated with vehicle; open circles, controls; filled triangles, rats with CIA treated with 3.0 μg of anti-IL-23 antibody; filled diamonds, rats with CIA treated with 6.0 μg of anti-IL-23 antibody. Means ± SD are shown. ^#^*P *= 0.021 versus controls, ^##^*P *= 0.021 versus controls, ^¶^*P *= 0.014 versus vehicle, ^§^*P *= 0.007 versus vehicle (Mann–Whitney *U *test). **(b) **Arthritis scores of control rats, vehicle-treated rats with CIA, and rats with CIA that were treated with anti-IL-23 antibody, from day 0 to day 28. Open squares, rats with CIA treated with vehicle; open circles, controls; filled triangles, rats with CIA treated with 3.0 μg of anti-IL-23 antibody; filled diamonds, rats with CIA treated with 6.0 μg of anti-IL-23 antibody. Means ± SD are shown. ^#^*P *= 0.021 versus controls, ^##^*P *= 0.021 versus controls (Mann–Whitney *U *test).

Figure 6a–c shows the paws of rats on day 28. Joint swelling in paws increased more in affected rats treated with vehicle than in controls (Figure 6a,b). Joint swelling in paws was reduced by IL-23 blockade in rats treated from day 14 (Figure 6b,c). Synovial tissues obtained on day 28 were stained with hematoxylin and eosin; active synovitis and bone erosion were detected in specimens from arthritic paws treated with vehicle (Figure 6e) but not in specimens from paws from controls (Figure 6d). The 3.0 μg dosage of anti-IL-23 antibody reduced inflammatory changes in synovial tissues of paws from rats treated from day 14 (Figure 6f). Paw volume increased progressively in rats without CIA (controls) from day 0 (open circles in Figure 7a). All arthritic animals groomed themselves well and maintained original body weight throughout the course of the disease. Paw volume was significantly greater in arthritic rats treated with vehicle than in control rats on days 18 and 21 (open squares in Figure 7a; *P *= 0.038 and 0.007, respectively). Maximum paw volume was found in rats with CIA treated with vehicle on day 21 (Figure 7a). Arthritis score was significantly greater in arthritic rats treated with vehicle than in control rats on days 18 and 21 (open squares in Figure 7b, *P *= 0.007 and 0.001, respectively). The Maximum arthritis score was found in rats with CIA that were treated with vehicle on day 28 (Figure 7b).

### Effect of anti-IL-23 antibody treatment on joint swelling

Joint swelling in paws was reduced in rats that were treated with IL-23 blockade from day 14. The 3.0 μg (filled triangles in Figure 7a) or 6.0 μg (filled diamonds in Figure 7a) dosage of anti-IL-23 antibody significantly reduced paw volume in rats treated from day 14 compared with rats treated with vehicle on day 21 (Figure 7a; *P *= 0.005 and 0.004, respectively). The 3.0 μg (filled triangles in Figure 7b) or 6.0 μg (filled diamonds in Figure 7b) dosage of anti-IL-23 antibody reduced the arthritis score in rats treated from day 14 compared with rats treated with vehicle on day 21 (Figure 7b; *P *= 0.069 and 0.77, respectively); however, there were no significant differences between rat groups.

### Effect of anti-IL-23 antibody treatment on joint damage as assessed by radiology

**Figure 8:**
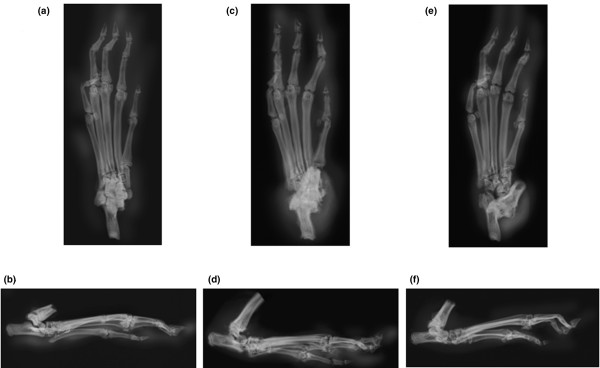
Typical radiographic images obtained on day 28, showing differences in paws. The groups were control rats, vehicle-treated rats with collagen-induced arthritis (CIA), and rats with CIA treated with anti-IL-23 antibody on day 28. **(a, c, e) **Frontal views; **(b, d, f) **lateral views. **(a, b) **Control rats. **(c, d) **Rats with CIA treated with vehicle. **(e, f) **Rats with CIA treated with anti-IL-23 antibody (3.0 μg) from day 14. Note that rats wth CIA treated with vehicle showed radiographic changes characterized by bone erosion and joint space narrowing.

Figure 8 shows typical radiographs of left limbs in each group obtained on day 28. Radiographic joint damage including bone erosion and loss of joint space was detected in X-rays of paws of rats with CIA treated with vehicle (Figure 8c,d), but not from paws of controls (Figure 8a,b). Compared with arthritic rats treated with vehicle, the 3.0 μg dosage of anti-IL-23 antibody reduced bone erosion changes and maintained joint space in arthritic rats treated from day 14 (Figure 8e,f).

## Discussion

This is the first report to demonstrate that IL-23 stimulates human osteoclast differentiation and that neutralizing IL-23 activity after the onset of CIA in rats has therapeutic potential. In the present study we showed that IL-23 directly induced human osteoclastogenesis in cultures of PBMC in the absence of osteoblasts or exogenous sRANKL. We also investigated whether IL-23 induces osteoclastogenesis from human monocytes alone; however, rhIL-23 did not do so (data not shown). In human immune cells, IL-23 receptors are expressed on activated or memory T cells, on natural killer cells, and, to a smaller extent, on macrophages and dendritic cells [9]. We therefore concluded that T cells are important in IL-23-induced human osteoclastogenesis from PBMC.

To clarify the factors involved in IL-23-induced osteoclastogenesis from PBMC, we used various inhibitors such as OPG (a decoy receptor for RANKL), anti-IL-17 antibody, and etanercept (a TNF-α blocking agent). All inhibitors clearly suppressed IL-23-induced osteoclastogenesis, even at 1.0 ng/ml, which was the most effective concentration of IL-23 in inducing osteoclastogenesis. These results indicate that cytokines, RANKL [4], IL-17 [21] and TNF-α [22] are, at least in part, associated with pathogenesis of RA manifested as IL-23-induced osteoclastogenesis.

To further clarify the mechanism of IL-23-induced osteoclastogenesis, we measured two major inflammatory cytokines that strongly affect osteoclastogenesis, namely IL-17 and IFN-γ. IL-17 promotes human osteoclastogenesis *in vitro *via the RANK–RANKL system or TNF-α [21,22]. We also showed that IL-17 induces human osteoclastogenesis from human monocytes even in the absence of osteoblastic cells or sRANKL through both inductively expressed TNF-α and constitutively expressed RANKL on human monocytes [22]. The synergistic effect of TNF-α and RANKL is important in this osteoclastogenesis; each cytokine was present at too low an expressed level to induce osteoclastogenesis individually [22].

In contrast, IFN-γ strongly inhibits osteoclastogenesis in humans and mice even at low concentrations [26,28]. We demonstrated that IL-23 induced IL-17 and IFN-γ production in human activated T cells, findings in accordance with those of the previous report by vanden Eijinden and colleagues [11]. Stimulation of T cells in our experiment was, however, much weaker than reported in that paper: we activated human T cells by using anti-CD3 antibody (0.1 μg/ml) and anti-CD28 antibody (2 μg/ml). In contrast, vanden Eijinden and colleagues activated human T cells by using anti-CD3 antibody (5 μg/ml) and anti-CD28 antibody (1 μg/ml), respectively. Our findings indicate that human T cells activated by mild stimulation can produce IL-17 and IFN-γ in response to rhIL-23.

Furthermore, we found that IL-23 induced IL-17 production, but not IFN-γ production, in a dose-dependent manner from human activated T cells, although there were no significant changes in IL-17 production levels by activated T cells after stimulation with IL-23. Considering the inductive effect of IL-17 and the inhibitory effect of IFN-γ as described above, however, we speculate that the balance of these two cytokines is important. Then we showed that the ratio of production levels of IL-17 to those of IFN-γ was elevated at 1 to 10 ng/ml IL-23; it is possible that the higher the ratio of IL-17 to IFN-γ, the greater the number of osteoclasts induced by IL-23. Although further studies are needed to clarify the contribution of other cytokines, for example IL-10 [11], released from T cells activated by IL-23, our findings suggest that the balance between IL-17 and IFN-γ may be important in IL-23-induced osteoclastogenesis.

In the present study, we also showed that IL-23 is important in inflammation and bone destruction at a later stage, after the onset of CIA, in rats. Anti-IL-23 antibody administered at the later stage of clinical arthritis significantly reduced paw volume in affected rats in a dose-dependent manner. In addition, radiographic and histologic analyses revealed that anti-IL-23 antibody tended to inhibit bone destruction in rats with CIA. In contrast, IL-23 has been reported to be important in the onset of arthritis in a model using IL-23 (p19)-deficient knockout mice [16]. In the present study, even at a later stage of CIA in rats, neutralization of endogenous IL-23 with anti-IL-23 antibody significantly reduced paw volume and inhibited bone destruction, indicating that IL-23 is necessary to induce inflammation and osteoclastogenesis even after the onset of clinical arthritis.

Staining of synovial tissues with hematoxylin and eosin showed that treatment with a low dose (3.0 μg) of anti-IL-23 antibody markedly reduced inflammatory changes and cartilage and bone destruction in paws, in contrast with findings in rats treated with vehicle. The 3.0 μg or 6.0 μg dosage of anti-IL-23 antibody reduced arthritis score in rats treated from day 14; however, there were no significant changes between rats treated with vehicle and those treated with anti-IL-23 antibody. The reason for these findings is unclear but may be related to the small number of rats used in this study. With regard to arthritis score, the effect of treatment with a high dose (6.0 μg) of anti-IL-23 antibody was smaller than that of treatment with a low dose (3.0 μg) of anti-IL-23 antibody. We speculate that antibodies against anti-IL-23 antibody might be produced in rats. In other words, a low dose of anti-IL-23 antibody may not induce the production of antibodies against anti-IL-23 antibody.

In this study, radiographic analyses revealed that treatment with anti-IL-23 antibody after the onset of CIA inhibited the progression of X-ray changes including joint space narrowing and bone erosion. A previous report revealed that expression of proinflammatory cytokines including TNF-α, IL-1β, and IL-6 is markedly reduced in IL-23-deficient (IL-23 p19^-/-^) mice [16]. These cytokines promote bone resorption by inducing differentiation of osteoclasts [2,6,7]. These findings therefore suggest that *in vivo *blockade of endogenous IL-23 has an effect on preventing bone destruction through a decrease in inflammatory cytokines associated with bone destruction. Taken together, our findings also indicate that anti-IL-23 antibody could reduce not only inflammation but also bone destruction.

The *in vivo *function of IL-23 remains to be determined; however, recent reports show that IL-23 stimulation can lead to the generation of an alternative T helper cell subset characterized by expression of high levels of the proinflammatory cytokine IL-17, but only low amounts of IFN-γ [10,11,16,18,29,30]. This novel T cell population was described as 'TH_IL-17._' A recent report by Langrish and colleagues [18] emphasizes the role of TH_IL-17 _cells in an animal model of EAE. In this report, the authors showed that IL-23-dependent TH_IL-17 _cells drive autoimmune inflammation in the brain and the neutralization of soluble IL-17 by using antibodies that partly protected the experimental mice from EAE. In the animal model of CIA, the resistance of IL-23 knockout mice was found to depend on the absence of IL-17-producing T helper cells, but not on an impaired Th1 immune response, demonstrating again the possible role of TH_IL-17 _cells in chronic and autoimmune inflammation [16]. A possible explanation for the effect of anti-IL-23 antibody on CIA is therefore that the number of IL-17-producing T cells might be reduced by IL-23 blockade.

In contrast, it has been shown with the use of an EAE model that adoptive transfer of IL-23-induced, IL-17-producing effector cells induces disease, whereas IL-12-induced, IFN-γ-producing effector cells do not [18]. Moreover, treatment with anti-IL-17 reduced disease severity in this study, whereas treatment with anti-IFN-γ exacerbated disease. Similarly, studies with IFN-γ-deficient and IFN-γ R-deficient mice have shown that IFN-γ and Th1 cells are not necessary for the development of autoimmunity in both EAE and CIA [31,32]. Furthermore, Park and colleagues recently reported that IFN-γ, IL-12, and IL-4 strongly inhibit the generation and population expansion of IL-17-expressing cells and their cytokine expression [33]. These studies suggest that at least IFN-γ may actually have a protective function. Thus, in view of the protective function of IFN-γ, the effect of anti-IL-23 antibody in our model of CIA may have been to normalize the ratio of IL-17 to IFN-γ; the IL-17 level was decreased, whereas that of IFN-γ did not change.

**Figure 9:**
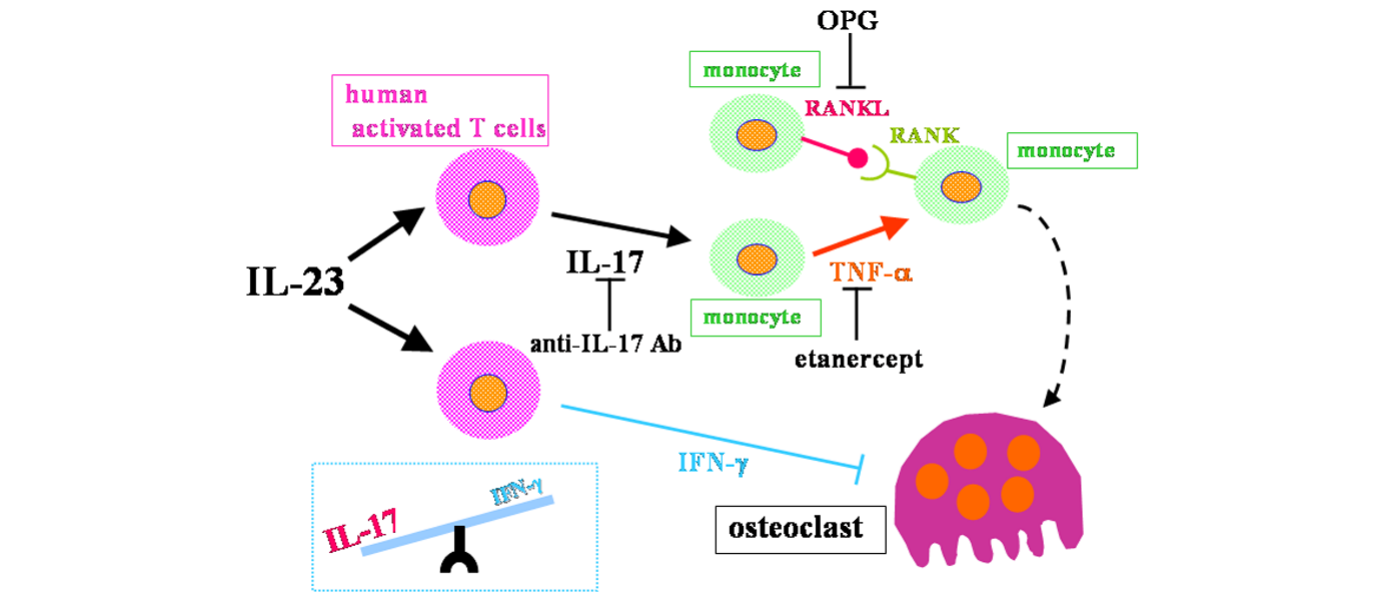
Mechanism of effect of IL-23 on human osteoclastogenesis. In a culture of human peripheral blood mononuclear cells, IL-23 directly induced osteoclastogenesis even in the absence of exogenous soluble receptor activator of NF-κB ligand (sRANKL). IL-17 induced by IL-23 from human activated T cells is the crucial cytokine for osteoclastogenesis through the mechanism of the RANK–RANKL system and TNF-α; IL-17 acts on monocytes, resulting in TNF-α production, inducing the differentiation of monocytes into osteoclasts by cooperating with RANKL, although RANKL alone expressed on monocytes does not induce osteoclastogenesis by binding to RANK. Furthermore, considering the inductive effect of IL-17 and the inhibitory effect of IFN-γ, it is speculated that the higher the ratio of IL-17 to IFN-γ, the greater the number of osteoclasts induced by IL-23. Ab, antibody; OPG, osteoprotegerin.

In our *in vitro *study, we showed that IL-23 induced key cytokines such as IL-17 and IFN-γ and that IL-23 induced human osteoclastogenesis via these cytokines (Figure 9). Our findings indicate that loss of the IL-17/IFN-γ balance leads to enhanced osteoclastogenesis induced by IL-23. Moreover, we speculate that IL-17, produced by T cells stimulated with IL-23, acts on monocytes, resulting in TNF-α production and inducing the differentiation of monocytes into mature osteoclasts through interaction with RANKL. In addition, the RANK–RANKL pathway expressed on monocytes induces osteoclast differentiation by binding to RANK. It has been reported that the gene expression of TNF-α is enhanced in synovial tissue of patients with RA [34] and that TNF-α accumulates in synovial fluids from patients with RA [35]. In addition, we and other groups have detected RANKL in synovial tissue and fluids from patients with RA [3,36,37]. From a clinical point of view, we believe that the levels of IL-17 induced by IL-23 in our experiment would significantly affect osteoclastogenesis in patients with RA because the concentrations of IL-17 in synovial fluid and culture medium of synovial tissue obtained from patients were less than 14 pg/ml and 40 pg/ml, respectively, in a previous study [21]. Thus, beginning with IL-23, the combined effects of TNF-α, RANKL, and IL-17 levels, all of which are elevated in patients with RA, contribute to osteoclastic bone resorption.

## Conclusion

In the present experiments, IL-23 directly induced osteoclastogenesis in cultures of human PBMC. Our findings strongly suggest that IL-23 is important in joint inflammation and bone destruction during the effector phase of CIA via the IL-23–IL-17 pathway and that IL-23 is an attractive target for the treatment of destructive arthritis. Furthermore, controlling the expression of IL-23 in patients with RA may provide a new treatment for inhibition of inflammation and bone destruction.

## Abbreviations

CIA = collagen-induced arthritis; CII = type II collagen; ELISA = enzyme-linked immunosorbent assay; EAE = experimental autoimmune encephalomyelitis; IFN = interferon; IL = interleukin; mAb = monoclonal antibody; M-CSF = macrophage-colony stimulating factor; MEM = minimal essential medium; OPG = osteoprotegerin; PBMC = peripheral blood mononuclear cells; RA = rheumatoid arthritis; RANKL = receptor activator of NF-κB ligand; rhIL-23 = recombinant human IL-23; sRANKL = soluble RANKL; Th1 = T helper type 1; TNF = tumor necrosis factor; TRAP = tartrate-resistant acid phosphatase.

## Competing interests

The authors declare that they have no competing interests.

## Authors' contributions

TY conducted the experimental work, performed the statistical analysis and drafted the manuscript. YN, MK, TF, TK, and NK helped with some experimental work. SK designed and conceived the study, coordinated the project and drafted the manuscript. All authors read and approved the final manuscript.

## Supplementary Material

Additional file 1 A TIFF file showing Osteoclast formation from human monocytes alone with adding rhIL-23. Click here for file
