# Supplementary figures and images for: IL-23 induces human osteoclastogenesis via IL-17 in vitro, and anti-IL-23 antibody attenuates collagen-induced arthritis in rats
Source: Arthritis Res Ther. 2007 Sep 23;9(5):R96. doi: 10.1186/ar2297 (PMC2212562; doi:10.1186/ar2297)

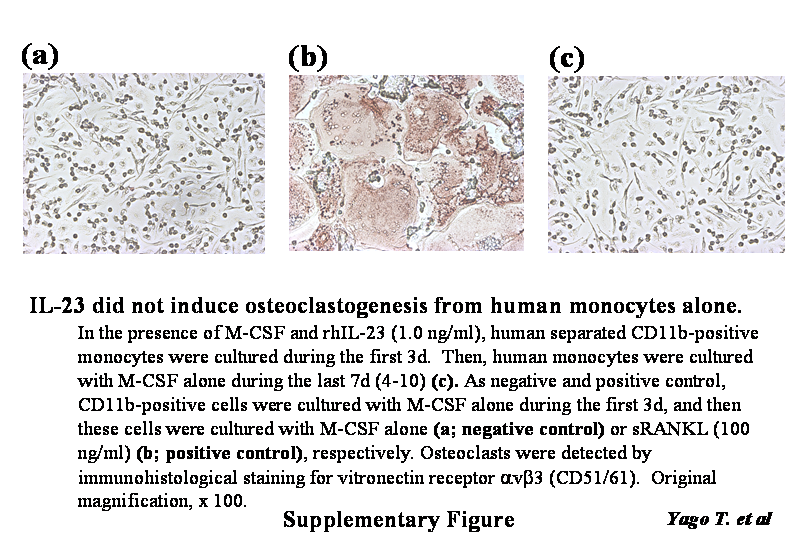

Supplement: Additional file 1 — A TIFF file showing Osteoclast formation from human monocytes alone with adding rhIL-23. [file ar2297-S1.tiff]
